# Supplementary material for: Cross-sectional analysis of UK research studies in 2015: results from a scoping project with the UK Health Research Authority
Source: BMJ Open. 2018 Oct 17;8(10):e022340. doi: 10.1136/bmjopen-2018-022340 (PMC6196875; doi:10.1136/bmjopen-2018-022340)
Supplement: Author's manuscript [file bmjopen-2018-022340.draft_revisions.pdf]

BMJ Open is committed to open peer review. As part of this commitment we make the peer review history of every article we publish publicly available.

When an article is published we post the peer reviewers' comments and the authors' responses online. We also post the versions of the paper that were used during peer review. These are the versions that the peer review comments apply to.

The versions of the paper that follow are the versions that were submitted during the peer review process. They are not the versions of record or the final published versions. They should not be cited or distributed as the published version of this manuscript.

BMJ Open is an open access journal and the full, final, typeset and author-corrected version of record of the manuscript is available on our site with no access controls, subscription charges or pay-per-view fees (http://bmjopen.bmj.com).

If you have any questions on BMJ Open's open peer review process please email info.bmjopen@bmj.com

BMJ Open

A cross-sectional study of UK trials in 2015: results from a scoping project with the UK Health Research Authority

Journal:	*BMJ Open*
Manuscript ID	bmjopen-2018-022340
Article Type:	Research
Date Submitted by the Author:	14-Feb-2018
Complete List of Authors:	Clark, Tim; Ludwig-Maximilians University, Institut für Medizinische Informationsverarbeitung, Biometrie und Epidemiologie (IBE), Faculty of Medicine<br>Wicentowski, Richard; Swarthmore College, Computer Science Department<br>Sydes, Matt; University College London, MRC Clinical Trials Unit, Institute of Clinical Trials and Methodology
Keywords:	Clinical trials < THERAPEUTICS, STATISTICS & RESEARCH METHODS, Protocols & guidelines < HEALTH SERVICES ADMINISTRATION & MANAGEMENT

SCHOLARONE™
Manuscripts

For peer review only

Title: A cross-sectional study of UK trials in 2015: results from a scoping project with the UK Health Research Authority

Running Head: Research in the UK

Version + Date: Version 1.04; (14-Feb-2018)

Tables/Figures: Tables=5

Figures=0

Name	Affiliation & IDs
Tim Clark	Institut für Medizinische Informationsverarbeitung, Biometrie und Epidemiologie (IBE), Faculty of Medicine, Ludwig-Maximilians University, Munich, Germany
Richard H Wicentowski	Computer Science Department, Swarthmore College, Swarthmore, PA, USA<br><br>ResearcherID: D-1051-2018<br>ORCID: 0000-0002-1187-9466
Matthew R Sydes	MRC Clinical Trials Unit at UCL, Institute of Clinical Trials and Methodology, UCL, London, UK<br><br>ResearcherID: C-3373-2008<br>ORCID: 0000-0002-9323-1371

Correspondence: Matthew Sydes m.sydes@ucl.ac.uk

Research support: This research received no specific grant from any funding agency in the public, commercial or not-for-profit sectors.

M Sydes acknowledges support from the UK Medical Research Council: MC_UU_12023/24

For peer review only

For peer review only - http://bmjopen.bmj.com/site/about/guidelines.xhtml

Word Count:

Section	V1.00 (by section)
Abstract	281
Introduction	442
Methods	713
Results	823
Discussion	1139
Total	3117

(Word counts include headings and reference numbers)

For peer review only

ABSTRACT

Objectives: To determine whether data on clinical trials held by the UK Health Research Authority (HRA) could be summarised automatically with minimal manual intervention. There are numerous initiatives to reduce research waste by improving the design, conduct, analysis and reporting of clinical trials. However, quantitative data on the characteristics of clinical trials and the impact of the various initiatives are limited.

Design: Feasibility study, using 1 year of data

Setting: We worked with the HRA on a pilot study using clinical trial applications submitted for UK-wide ethical review. We extracted into a single dataset, information held in anonymised XML files by the Integrated Research Application System (IRAS) and the HRA Assessment Review Portal (HARP). Applications for clinical trials from 2014-2016 were provided. We used standard text extraction methods to assess information held in free-text fields. We use simple, descriptive methods to summarise the research activities that we extracted.

Participants: Not applicable -- records-based study

Interventions: Not applicable

Primary and secondary outcome measures: Feasibility of extraction and processing

Results: We successfully imported 1,775 non-duplicate trial applications from the XML files into a single database. Of these, 963 were randomised controlled trials, 812 were other studies. Most studies received a favourable opinion. There was limited patient and public involvement (PPI) in the studies. Most, but not all, studies were planned for publication of results. Novel clinical trial designs (e.g. adaptive and Bayesian designs) were infrequently reported.

Conclusions: We have demonstrated that the data submitted from IRAS to the HRA and its HARP system are accessible and can be queried for information. We strongly encourage the development of fully-resourced collaborative projects to further this work. This would aid understanding of how study characteristics change over time and across

For peer review only

therapeutic areas, as well as the progress of initiatives to improve the quality and relevance of clinical trials.

For peer review only

For peer review only - http://bmjopen.bmj.com/site/about/guidelines.xhtml

ARTICLE SUMMARY

Strengths and limitations of this study

- First study to draw information from HRA's HARP system into one searchable dataset
- Anonymised data from the HRA's HARP system can be interrogated with minimal manual intervention
- Feasibility study, so limited to data from 2015 only and excluding Phase I commercial studies
- Aligning the questions from the XML document was not straightforward.
- The search terms chosen for the free-text fields were not exhaustive and because it was unfeasible in a pilot to review all applications, the sensitivity and specificity of the text-mining methodology could not be calculated

For peer review only

INTRODUCTION

The need to improve the quality of clinical research increasingly understood.[1] With a particular focus on clinical trials, the "gold standard" for evidence-based medicine, there has been a noticeable push towards: improving study protocols;[1] developing and implementing newer methodologies, such as adaptive designs;[2] involving patients in the design, conduct and management of studies;[3] and ensuring that study results are quickly and accurately reported.[4]

In order to properly evaluate the current state of clinical research and changes over time, it is clearly necessary to have unfettered access to the research protocols and study results.[5,6] There are a number of limitations in accessing such information. Published evaluations of the state of clinical research are mainly based on the clinical study publication due to the difficulty in obtaining access to unpublished research protocols.[7] Research registers are incomplete for many jurisdictions and/or are, like study publications, limited in detail compared to the research protocol. For example, clinicaltrials.gov[8] has good coverage of clinical trials in North America where registration is mandatory and the WHO International Clinical Trials Registry Portal[9] provides access to 15 different regional or national registries, but both have markedly less detail than in each study's research protocol. Published evaluations of the literature are likely affected by publication bias and there are often discrepancies between research publications and their underpinning protocols.[10]

The ability to analyse a large database of application forms, each of which contain very detailed information taken directly from the research protocol, would enable researchers to perform a detailed examination of the characteristics of clinical trials, particularly those necessary for generating reliable evidence. This would aid understanding of how study characteristics change over time and across therapeutic areas, as well as the progress of initiatives to improve the quality and relevance of clinical trials.[11-15] Therefore, we approached the UK Health Research Authority (HRA) and, with their

For peer review only

For peer review only - http://bmjopen.bmj.com/site/about/guidelines.xhtml

support and having signed the appropriate confidentiality declarations, developed a pilot project to determine the feasibility of interrogating with minimal manual intervention the data contained in HARP (HRA Assessment Review Portal); HARP is a web based management information system used by the HRA for all research ethics applications that require NHS REC (National Health Service Research Ethics Committee) review and/or HRA Approval. We negotiated access to data on clinical trials submitted in 2015. The extract had personal data and organisational identifiers removed by HRA and did not include phase I commercial trials.

Our pilot focused on automatically summarising the characteristics of clinical trials, including therapeutic area, blinding, randomization, use of Independent Data Monitoring Committee (IDMC), patient and public involvement (PPI) in the research, dissemination of the trial results, and use of new methodologies such as adaptive designs.

For peer review only

For peer review only - http://bmjopen.bmj.com/site/about/guidelines.xhtml

METHODS

The UK Health Research Authority (HRA) collects applications for Research Ethics Committee “approval” (a favourable opinion) through the Integrated Research Application System (IRAS) and stores them in HARP. IRAS is a web-based system used to capture the information required by review bodies in the UK, including the Medicines and Healthcare Products Regulatory Agency and HRA. When the forms are completed, IRAS saves the results as an XML document that can be used to generate PDF copies of the required forms and to repopulate the online web tool so users can edit their applications.

Once the appropriate permissions were agreed, the HRA provided us with anonymised applications for clinical trials submitted for review from 2014-2016, excluding commercial phase I and healthy volunteer studies. For this pilot, we wished to focus on the extraction of clinical study characteristics of interest from the PDF versions of the HRA forms underlying the IRAS application system. We were provided with an incomplete mapping between the questions on PDF and the data stored in the XML document, such that many questions relevant to our study were not immediately labelled; nor was it apparent which questions were effectively switched on and off for applicants in completing the system’s initial filter questions for their application. Therefore, we reverse-engineered the structure of the data in IRAS to determine which XML tags were responsible for creating the content we wanted to extract from the XML files to study.

The IRAS tool has been updated over time; not all of applications were created using the same version. Although the majority (91%) of the XML documents were created with one version (the latest version of the tool in the dataset), 8 different versions were present in the dataset we received. We were not provided with the documentation detailing the ways in which the versions differed; we had access only to the latest version. Our inspection of the data indicated that the differences relevant to this pilot

For peer review only - http://bmjopen.bmj.com/site/about/guidelines.xhtml

study were minor and we corrected for these when they were detected; however, there may be some undetected differences where the distinction between versions was important for our study.

For our pilot project, we chose to collect information on: the number of randomised controlled trials (RCT); the trial phase; the therapeutic area; the number of clinical trials of investigational medicinal products (CTIMPs – trials that require registration with regulatory body); the research method used (e.g. RCT, feasibility/pilot study, blinding); the use of blinding; the use of systematic or formal literature reviews in planning the study; the use of adaptive or Bayesian designs; the use of a data monitoring committee; the plans for dissemination of findings; and the role of patient and public involvement (PPI) in aspects of the research. Success would be measured in terms of our ability to access the information and generate descriptive summaries with minimal need for manual data reviews.

The data for our target areas were either held in multiple choice questions that we could identify and summarize or in free-text responses from which information needed to be determined and extracted. For those questions, we pre-processed the text using Spacy (https://spacy.io/), a natural language processing (NLP) package for Python. This involved identifying sentence boundaries and performing morphological analysis to convert words into their canonical dictionary form. We then used regular expressions to match phrases we were interested in, ignoring differences relating to spacing, hyphenation and capitalization; roman numerals were standardized to Latin numbers. Search terms are shown in **Table 1**. For example, when looking for evidence that a systematic review was either performed or an existing review used during the trial planning process, we searched for phrases such as “systematic review”, “reviewed systematically”, “literature review”, “evidence review” and “evidence-based review” in the answers provided to the relevant questions on the form. For adaptive designs, we employed the US Food and Drug Administration (FDA) definition, namely “a study that

For peer review only - http://bmjopen.bmj.com/site/about/guidelines.xhtml

154 includes a prospectively planned opportunity for modification".[19] We extracted the sentence containing the target phrase as well as one sentence before and after into a separate document. These textural extracts were then reviewed by the authors to identify true and false positives.

The data extracted from the separate XML files were collated into one dataset and descriptive analyses of this dataset were done with Stata (College Station, TX).

For peer review only

RESULTS

We received (30-Jun-2016) the XML files for 1,814 application records submitted for ethical review during the period specified, and extracted by HRA from the IRAS system. Of these, 1659 (92%) were from 2015, 154 (9%) from 2014, and 1 was from 2016. Three records were corrupted and could not be processed. In discussion with HRA, we discarded some records to remove duplicates. We kept one of 4 entries for the WHEAT trial which had been sent to multiple committees as part of research on consistency of Research Ethics Committee (REC) opinions.[16] We discarded one of two entries for a further study, which was initially given a favourable opinion and then submitted additionally to a specialist REC in Scotland (again, favourably). Thirty-two further records were discarded that initially had an unfavourable opinion before re-submission as a (near) identical, separate application for review. We discarded the first applications and kept the re-submissions, regardless of the subsequent review's outcome. Therefore, our dataset included 1,775 studies.

The filter questions switched off, for some applications, questions in which we were interested. For example, trial phase was only recorded for CTIMPs and the use of data monitoring committees was infrequently recorded. Furthermore, the use of blinding was not sufficiently well captured in the system to allow us to present reliable data.

A total of 963/1775 (54%) of the applications were stated as being randomised controlled trials (RCT). **Table 2** describes the disease setting, research method, CTIMP and REC opinion by whether they were an RCT. The most common research area, using the categorisation of the IRAS system, was cancer, followed by cardiovascular, musculoskeletal and respiratory diseases; around one third of records specified more than one area. This is broadly consistent with previous reviews.[17,18] Around one fifth of the RCTs was a pilot study; one third of records were employing more than one method. The REC gave a favourable opinion to the vast majority of applications (1740/1775, 98%), but required additional information for most of these 1323/1740 (75%).

For peer review only - http://bmjopen.bmj.com/site/about/guidelines.xhtml

Over half of the RCTs (515/963, 53%) were CTIMPs, of which 315/515 (61%) were CTIMPs of unlicensed products, 146/515 (28%) licensed CTIMPs in a new setting and 66/515 (13%) CTIMPs used according to their summary of product characteristics (SmPC); the categories were not mutually exclusive. Over a third (284/812, 35%) of the other studies (not RCTs) were also CTIMPs.

Table 3 describes the plans in design and dissemination. Most RCTs (895/963, 93%) were not recorded as being preceded by a formal systematic review of the literature. Only 15 (2%) RCTs were detectably designed under a Bayesian framework; 20 (2%) were detectable as having an adaptive design. There were 34 cluster randomised and 5 stepped wedge trials, overall. 614 (63%) RCTs were requesting new biological samples; 120 (12%) RCTs were seeking access to previous biological samples.

The large majority of research (1667/1775, 94%) was planned for dissemination in the peer-reviewed literature and most (1585/1775, 89%) were planned for conference presentations. A total of 475/963 (49%) RCTs and 352/812 (43%) other studies were planned for submission to regulatory authorities. A small number, 114/1775 (6%) were planning to offer raw data to external applicants as a key form of dissemination.

Table 4 notes the reported involvement of patients and the public (PPI) using the categories specified by IRAS. 726/1775 (41%) of studies claim PPI involvement in the design and 659/1775 (37%) planned PPI involvement in dissemination of the findings. PPI engagement in undertaking and management of the research was less common, and few studies (147/1775, 8%) planned PPI involvement in analysis. Around one third of studies involve PPI in two or more of these IRAS-defined areas.

Table 5 shows within clinical trials the similarities and differences across the phase of research. Trial phase was asked as a series of separate yes/no questions for phase I, II,

For peer review only

III and IV. These were not mutually exclusive. For the purpose of summarising, we selected the highest level if more than one option was selected. Early phase trials were more likely to be designed within a Bayesian framework; phase III trials most often reported the use of a placebo and the use of an Independent Data Monitoring Committee; phase IV trials were least likely to be submitted to a regulatory authority or put on a website, but most often reported PPI involvement in all 5 broad areas.

Table 1 shows the outcome of the manual review of the extracted free-text responses. Accuracy, defined as number of true positives divided by overall total ranged from 43% for adaptive designs to 100% for stepped wedge. The false positives related to studies described as "phase 1/2" or "phase 2/3", but with no evidence of an adaptive step, e.g. Phase 1/2 was often used to describe a classical pharmacokinetic study in patients rather than healthy volunteers; the database did not contain healthy volunteer studies. For "systematic review" and "meta-analysis" the search often picked up references to previous studies and not the planned study.

For peer review only

DISCUSSION

We achieved our primary feasibility aim of negotiating access to the centralised UK approvals system and devising a way of extracting information from a series of separate XML files. We anticipate that the programmes we developed could extract further pertinent points of information with minimal manual involvement. We demonstrated that we successfully extracted data by achieving our primary descriptive aim of systematically reporting on the state of clinical trials research in the UK over one year, focused on 2015.

We found that nearly a thousand RCTs were submitted for approval in the UK during that year, and more than 800 other studies. A key message is the volume of research activity in the UK, with the country demonstrably research active. The majority of applications received a favourable opinion but most required further information first, suggesting that applications could be better completed, saving time and effort for all parties.

It was notable that the reported use of adaptive designs was low and was not markedly different from earlier estimates.[17,18] This is disappointing, as many members of this design family have scope to reduce time to answer and/or reduce the average cost per answer, particularly by moving away early from treatment approaches that are not likely to improve outcomes sufficiently for patients.[20] However, Bayesian approaches have penetrated 10% of early phase CTIMPs. The reported use of Independent Data Monitoring Committees (IDMCs) for phase III CTIMPs was, perhaps, low at 58% but this is a substantial increase over the prevalence in trials results papers published in high impact-factor journals in 1990 and 2000.[21] Involvement of patients and the public in various aspects of research is increasingly seen as important, but reported rates of engagement were quite low: more than half of studies had no PPI involvement.[22] Cursory review of the free-text question associated with these PPI categories suggests that some applicants may have been a little generous in choosing to select a particular category, suggesting that the numbers we report may be over-estimates. Other

For peer review only

comments suggest that PPI is not needed, perhaps reflecting that the value of PPI is yet to be recognised by some researchers.[3] Further research in this area is required.

Most but not all trials were planned for publication; there is still some way to go on this transparency aim. That raw data would be made available for some studies is encouraging and we hope that this will increase with time for appropriate projects from qualified researchers.[23]

Extracting the information from the HRA system was more effortful than expected. Moreover, important characteristics of clinical trials like blinding could not be analysed and certain search terms clearly had limitations. The issue of blinding is particularly important, as it is one of the desirable characteristics in generating reliable evidence from clinical trials.[11] However, now that we have completed this feasibility step, it is possible for future research projects to access an expanded dataset and work with the HRA to organise the database in a way that will facilitate its analysis. Access to several years of data would allow researchers, as well as the HRA itself, to examine the progress of initiatives to improve the quality of clinical research, such as those designed to encourage PPI and publication of clinical trial results.[13,15] We would also encourage the HRA to adopt standard definitions to describe the characteristics of clinical trials, as done in the USA.[25] Confusion over the definition of terms greatly complicates efforts to automatically analyse clinical research.

Our pilot work has some limitations. We had no straightforward way of aligning the questions in the PDF document with the information stored in the XML document. Perhaps if we had negotiated access to the backend software used by IRAS to generate these PDF forms, we may have been able to do this automatically; instead, we needed to reverse-engineer the IRAS system in order to figure out which XML tags were responsible for creating the content we wanted to study. Not all of the applications had been created using the same version of the IRAS tool. Although a large majority of the

For peer review only

documents were created using the latest version of the tool, 8 different versions were present in the dataset we received. We did not receive any guidance on the ways in which one version of the tool differed from another version, and we only had access to the latest version. We made efforts to determine the specific situations in which questions were not asked rather than asked but not answered, in order to make this distinction ourselves, though ideally this could be indicated directly in the XML dataset. The labelling of missing answers and inactivated questions was not consistent. There is scope for applicants to misunderstand the questions and misrepresent the data. For example, 3 trials were noted as using RCT methodology but answered "no" to the question: "Will participants be allocated to groups at random?" We checked the data and found these were not actually RCTs; we included them with the "other studies". Likely this error by the applicants activated or de-activated some questions inappropriately. In this example, the RCT method question actually asked about randomisation of individuals and these three applicants who submitted cluster randomised trials had answered this question inconsistently. Finally, the search terms chosen for the free-text fields were not exhaustive and because it was unfeasible in a pilot to review all applications, the sensitivity and specificity of the NLP package could not be calculated.

Some interesting information was held only in the free-text fields. We used standard methods to extract this information using automation supplemented by manual checking. There is a wealth of free-text information, which through the application of text-mining techniques, could provide incredibly valuable insights into the characteristics of health research. For this pilot study, we did not have the resources to review all of the questions and we particularly could not spend time reading free-text fields. Therefore, we felt obliged not to correct categorical questions if we noticed they were contrasted by free-text; however, a fully-resourced study could do this. For example, we noticed at least one entry, which ticked boxes for PPI engagement and expanded on this by claiming that, as doctors, they knew what patients wanted and did not need to trouble patients for their time on these aspects.

For peer review only - http://bmjopen.bmj.com/site/about/guidelines.xhtml

323

324 In conclusion, we have demonstrated that anonymised data from the HRA's research
325 system is accessible and can be queried for information. We strongly encourage the
326 development of fully-resourced collaborative projects to delve more deeply into this
327 data. We believe that it is imperative that the characteristics of clinical research in the
328 UK are understood, as these underpin clinical guidance. Finally, there are many ongoing
329 initiatives to improve the quality of clinical research, but only by fully understanding the
330 state of, and changes to, the research profile of the UK, can we appreciate their impact.
331

For peer review only

AUTHOR CONTRIBUTIONS

Grouped by role

Conceptualised the study – TPC, MRS; Designed the study – TPC, RHW, MRS; Collected the data – TPC, RHW, MRS; Analysed and interpreted the data – TPC, RHW, MRS; Wrote critical sections of manuscript – TPC, RHW, MRS; Reviewed and approved final manuscript – TPC, RHW, MRS

Grouped by author

TPC conceptualised the study, designed the study, collected the data, analysed and interpreted the data, wrote critical sections of manuscript, and reviewed and approved final manuscript. RHW designed the study, collected the data, analysed and interpreted the data, wrote critical sections of manuscript, and reviewed and approved final manuscript. MRS conceptualised the study, designed the study, collected the data, analysed and interpreted the data, wrote critical sections of manuscript, and reviewed and approved final manuscript.

For peer review only

DATA SHARING

The data used in this analysis were obtained from the Health Research Authority under a confidentiality agreement and is not under the researcher's gift to share. Applications for this data or expanded data must involve the HRA. Access to the processing code is available through the MRC Clinical Trials Unit at UCL's usual Data Release Request approach.

For peer review only

COMPETING INTERESTS

MRS = None

RHW = None

TPC = Worked as a consultant for the clinical research organisation ICON in the previous three years

For peer review only - http://bmjopen.bmj.com/site/about/guidelines.xhtml

ACKNOWLEDGEMENTS

Hazel Gage, Amanda Hunn, Janet Messer - UK Health Research Authority; Prof. Dr. Ulrich Mansmann, IBE, Munich, Germany.

For peer review only

TABLES AND FIGURES

Table 1: Manual review of data

Property	Example of search term*	True Positive	False Positive	Accuracy
**Systematic review**		**139**	**53**	**72.4%**
Systematic review	Systematic NEAR review	104	39	72.7%
Systematic review	Review NEAR systematically	2	0	100.0%
Systematic review	Literature NEAR review	29	13	69.0%
Systematic review	Evidence NEAR review	4	1	80.0%
Systematic review	Evidence-based review	0	0	.
**Meta-analysis**		**82**	**31**	**72.6%**
Meta-analysis	Meta-analysis	80	31	72.1%
Meta-analysis	Pooled analysis	2	0	100.0%
Meta-analysis	Integrated analysis	0	0	.
**Adaptive design**		**51**	**69**	**42.5%**
Adaptive design	Adaptive design	19	0	100.0%
Adaptive design	Seamless design	1	0	100.0%
Adaptive design	Sample size re-estimation	15	0	100.0%
Adaptive design	Multi-arm multi-stage	0	0	.
Adaptive design	Adaptive randomisation	8	0	100.0%
Adaptive design	Drop-the-loser	0	0	.
Adaptive design	Pick-the-winner	0	0	.
Adaptive design	Continual reassessment	2	0	100.0%
Adaptive design	Mams design	0	0	.
Adaptive design	CRM design	0	0	.
Adaptive design	Phase 1/2	4	44	8.3%
Adaptive design	Phase 2/3	2	25	7.4%
**Bayes**		**88**	**2**	**97.8%**
Bayes	Bayes	88	2	97.8%
**Cluster RCT**		**80**	**1**	**98.8%**
Cluster RCT	Cluster random	46	1	97.9%
Cluster RCT	Cluster RCT	26	0	100.0%
Cluster RCT	Randomised cluster	2	0	100.0%
Cluster RCT	Control cluster	6	0	100.0%
**Stepped wedge**		**17**	**0**	**100.00%**
Stepped wedge	Stepped NEAR wedge	16	0	100.0%
Stepped wedge	Step NEAR wedge	1	0	100.0%

*This is an example of the search term used. All variations of the spelling, capitalisation, hyphenation etc., were covered in the search terms used

For peer review only

For peer review only - http://bmjopen.bmj.com/site/about/guidelines.xhtml

Table 2: Overview of research activities submitted for REC approval

Characteristic	RCT N	RCT %	Other N	Other %	Total N	Total %
N	963		812		1775	
**Therapeutic area[1]**						
Cancer	168	17%	192	24%	360	20%
Cardiovascular	94	10%	94	12%	188	11%
Musculoskeletal	97	10%	30	4%	162	9%
Respiratory	97	10%	30	4%	159	9%
Paediatrics	56	6%	30	4%	148	8%
Neurological	73	8%	30	4%	145	8%
Mental Health	66	7%	30	4%	117	7%
Inflammatory & Immune System	72	7%	30	4%	113	6%
Oral and Gastrointestinal	64	7%	30	4%	113	6%
Blood	44	5%	67	8%	111	6%
Diabetes	58	6%	38	5%	96	5%
Infection	55	6%	30	4%	92	5%
Renal and Urogenital	39	4%	30	4%	82	5%
Generic Health Relevance	46	5%	30	4%	76	4%
Metabolic and Endocrine	32	3%	30	4%	70	4%
Dementias & Neurodegenerative	27	3%	36	4%	63	4%
Skin	36	4%	30	4%	57	3%
Reproductive Health & Childbirth	30	3%	30	4%	56	3%
Stroke	27	3%	30	4%	53	3%
Eye	24	2%	28	3%	52	3%
Injuries and Accidents	22	2%	30	4%	36	2%
Congenital Disorders	10	1%	20	2%	30	2%
Ear	6	1%	7	1%	13	1%
**Count of therapeutic areas claimed**						
None	52	5%	49	6%	101	6%
1	660	69%	495	61%	1155	65%
2	201	21%	200	25%	401	23%
3	29	3%	49	6%	78	4%
4 or more	21	2%	19	2%	40	2%
**Research methods used**						
Randomised controlled trial	963	100%	2	0%	965	54%
Feasibility/ pilot study	177	18%	300	37%	477	27%
Questionnaire	98	10%	139	17%	237	13%
Cohort observation	23	2%	161	20%	184	10%
Qualitative research	90	9%	76	9%	166	9%
Controlled trial, no randomisation	3	0%	157	19%	160	9%
Laboratory study	30	3%	47	6%	77	4%
Case series/ case note review	7	1%	45	6%	52	3%
Cross-sectional study	4	0%	31	4%	35	2%
Case control	5	1%	25	3%	30	2%
Database analysis	8	1%	17	2%	25	1%
Epidemiology	7	1%	5	1%	12	1%
Meta-analysis	0	0%	0	0%	0	0%
Other	68	7%	269	33%	337	19%
**Count of research methods used**						
1	641	67%	522	64%	1163	66%

For peer review only

For peer review only - http://bmjopen.bmj.com/site/about/guidelines.xhtml

Characteristic	RCT N	RCT %	Other N	Other %	Total N	Total %
2	192	20%	160	20%	352	20%
3	73	8%	97	12%	170	10%
4	48	5%	25	3%	73	4%
5	7	1%	7	1%	14	1%
6	2	0%	1	0%	3	0%
**CTIMP**						
Unlicensed	315	33%	189	23%	504	28%
New use	146	15%	70	9%	216	12%
Within SmPC	66	7%	26	3%	92	5%
Other	10	1%	5	1%	15	1%
Any of these	515	53%	284	35%		
**Involves ionising radiation**						
Yes	666	69%	582	72%	1248	71%
No	297	31%	222	28%	519	29%
Missing	0	n/a	8	n/a	8	n/a
**REC opinion**						
Favourable	942	98%	798	98%	1740	98%
Unfavourable	21	2%	14	2%	35	2%
Favourable	200	21%	217	27%	417	23%
Favourable (extra info)	742	77%	581	72%	1323	75%
Unfavourable	21	2%	14	2%	35	2%

For peer review only

Table 3: Issues in design and dissemination in all entries

Characteristic	RCT N	RCT %	Other N	Other %	Total N	Total %
N	963		812		1775	
**Review of data as part of development**						
Neither	895	93%	759	93%	1654	93%
Systematic review only	38	4%	32	4%	70	4%
Meta-analysis only	18	2%	13	2%	31	2%
Both	12	1%	8	1%	20	1%
**Design characteristics**						
Neither	926	96%	778	96%	1704	1%
Adaptive design	20	2%	9	1%	29	2%
Bayes design	15	2%	24	3%	39	2%
Both	2	0%	1	0%	3	0%
Cluster randomised	26	3%	8	1%	34	2%
Stepped wedge	3	0%	2	0%	5	0%
**Sample collection**						
Taking new samples	614	64%	422	52%	1036	58%
Accessing stored samples	120	12%	110	14%	230	13%
**Plans for dissemination**						
Peer reviewed scientific journal	912	95%	755	93%	1667	94%
Conference presentation	869	90%	716	88%	1585	89%
Internal report	641	67%	526	65%	1167	66%
Publication on website	536	56%	367	45%	903	51%
Submission to regulatory authority	475	49%	352	43%	827	47%
Other publication	239	25%	178	22%	417	23%
Access to raw data	191	20%	133	16%	114	6%
Other	0	0%	3	0%	324	18%
No plans to report or disseminate					3	0%

[1] Not asked for non-regulatory RCTs

For peer review only

378 **Table 4: Reported patient and public involvement**

Characteristic	RCT N	RCT %	Other N	Other %	Total N	Total %
N	963		812		1775	
**Areas of PPI activity[2]**						
Design of the research	403	42%	323	40%	726	41%
Dissemination of findings	375	39%	284	35%	659	37%
Undertaking the research	242	25%	219	27%	461	26%
Management of the research	225	23%	129	16%	354	20%
Analysis of results	72	7%	75	9%	147	8%
**Number of areas of PPI activity**						
None	389	40%	302	37%	691	39%
1	205	21%	209	26%	414	23%
2	139	14%	117	14%	256	14%
3	125	13%	93	11%	218	12%
4	66	7%	37	5%	103	6%
All	39	4%	32	4%	71	4%

379

For peer review only

Table 5: Characteristics of the CTIMPs by trial phase

Trial phase[2] / Characteristic	Ph I N	Ph I %	Ph II N	Ph II %	Ph III N	Ph III %	Ph IV N	Ph IV %	Total N	Total %
N – all entries	99		287		324		98		808	
**Design characteristics**										
Neither	86	89%	261	93%	303	94%	90	94%	740	93%
Adaptive	1	1%	7	2%	13	4%	3	3%	24	3%
Bayes	10	10%	12	4%	5	2%	2	2%	29	4%
Both	0	0%	2	1%	0	0%	1	1%	3	0%
Cluster randomised	0	0%	0	0%	0	0%	0	0%	0	0%
Stepped wedge	0	0%	0	0%	0	0%	0	0%	0	0%
Involves placebo	21	22%	123	44%	163	51%	25	26%	332	42%
**Independent Data Monitoring Committee**										
No	40	82%	84	59%	61	42%	46	78%	231	58%
Yes	9	18%	59	41%	84	58%	13	22%	165	42%
Missing	*8*		*139*		*176*		*37*		*400*	
**Methods of dissemination**										
Peer reviewed scientific journal	86	89%	265	94%	290	90%	93	97%	86	89%
Submission to regulatory authority	85	88%	215	76%	272	85%	48	50%	85	88%
Conference presentation	82	85%	253	90%	271	84%	89	93%	82	85%
Internal report	71	73%	212	75%	259	81%	60	63%	71	73%
Publication on website	56	58%	161	57%	203	63%	45	47%	56	58%
Other publication	26	27%	76	27%	92	29%	17	18	26	27%
Access to raw data	8	8%	12	4%	25	8%	8	8%	8	8%
Other	12	12%	47	17%	48	15%	18	19%	12	12%
No plans to report or dissemination	0	0%	0	0%	2	1%	0	0%	0	0%
**Areas of PPI activity[1]**										
Design of the research	26	27%	82	29%	39	12%	50	52%	26	27%
Management of the research	10	10%	34	12%	28	9%	31	32%	10	10%

For peer review only - http://bmjopen.bmj.com/site/about/guidelines.xhtml

Characteristic / Trial phase[2]	Ph I N	Ph I %	Ph II N	Ph II %	Ph III N	Ph III %	Ph IV N	Ph IV %	Total N	Total %
Undertaking the research	14	14%	47	17%	36	11%	29	30%	14	14%
Analysis of results	1	1%	15	5%	7	2%	12	13%	1	1%
Dissemination of findings	20	21%	74	26%	64	20%	44	46%	20	21%
None of the above	53	55%	149	53%	223	69%	35	36%	460	58%
**REC opinion**										
Favourable	97	98%	277	97%	318	98%	93	95%	785	97%
Unfavourable	0	0%	5	2%	3	1%	3	3%	11	1%
Favourable	14	14%	51	18%	40	12%	15	15%	120	15%
Favourable (after extra info)	83	84%	226	79%	278	86%	78	80%	665	82%
Unfavourable	0	0%	5	2%	3	1%	3	3%	11	1%

[1] Not mutually exclusive

[2] Calculated

For peer review only

For peer review only - http://bmjopen.bmj.com/site/about/guidelines.xhtml

REFERENCES

1. Ioannidis JP. Why Most Clinical Research Is Not Useful. PLoS Med 2016; 13(6): e1002049.

2. Hatfield I, Allison A, Flight L, Julious SA, Dimairo M. Adaptive designs undertaken in clinical research: a review of registered clinical trials. Trials 2016; 17(1): 150.

3. Elliott J. HRA Strategy for Public Involvement (v1.1). London, UK: Health Research Authority, 2013.

4. alltrials.net. alltrials.net (accessed 2013-05-24 2013).

5. English R, Lebovitz Y, Griffin R, Forum on Drug Discovery Development and Translation, Institute of Medicine. Transforming Clinical Research in the United States: Challenges and Opportunities: Workshop Summary. Institute of Medicine of the National Acadamies, Naitonal Acadamies Press; 2010. p. 151.

6. Chalmers I, Glasziou P, Godlee F. All trials must be registered and the results published. BMJ 2013; 346: f105.

7. Chan AW, Upshur R, Singh JA, Ghersi D, Chapuis F, Altman DG. Research protocols: waiving confidentiality for the greater good. BMJ 2006; 332(7549): 1086-9.8. ClinicalTrials.gov. www.clinicatrials.gov.

9. WHO Clinical Trials Registration Portal. http://www.who.int/ictrp/en/.

10. Chan AW, Hrobjartsson A, Jorgensen KJ, Gotzsche PC, Altman DG. Discrepancies in sample size calculations and data analyses reported in randomised trials: comparison of publications with protocols. BMJ 2008; 337: a2299.

11. Califf RM, Zarin DA, Kramer JM, Sherman RE, Aberle LH, Tasneem A. Characteristics of Clinical Trials Registered in ClinicalTrials.gov, 2007-2010. JAMA. 2012;307(17):1838–1847.

12. Grignolo A. The Clinical Trials Transformation Initiative (CTTI). Ann Ist Super Sanita 2011;47:14-8

13. INVOLVE (2015) Public involvement in research: values and principles framework, INVOLVE: Eastleigh

14. Chan A-W, Tetzlaff J, Altman D, Dickersin K, Moher D. SPIRIT: New guidance for content of clinical trial protocols. Lancet 2013;381:91-2.

15. Moorthy VS, Karam G, Vannice KS, Kieny M-P (2015) Rationale for WHO's New Position Calling for Prompt Reporting and Public Disclosure of Interventional Clinical Trial Results. PLoS Med 2015; 12(4): e1001819.

16. Gale C, Hyde MJ, Modi N, group Wtd. Research ethics committee decision-making in relation to an efficient neonatal trial. Arch Dis Child Fetal Neonatal Ed 2017; 102(4): F291-F8.

17. Clark T. Ursula Berger, U. Mansmann, U: Sample size determinations in original study protocols for randomised clinical trials submitted to UK research ethics committees: review. BMJ 2013, 346:f1135.

18. Clark T: Sample size calculations in clinical trials of investigational medicinal products: an analysis of ongoing practice. Der Andere Verlag; 2013.

19. FDA Draft Guidance for Industry, Adaptive Design Clinical Trials for Drugs and Biologicals, February 2010

20. Parmar MK, Barthel FM, Sydes M, et al. Speeding up the evaluation of new agents in cancer. J Natl Cancer Inst 2008; 100(17): 1204-14.

For peer review only - http://bmjopen.bmj.com/site/about/guidelines.xhtml

21. Sydes MR, Altman DG, Babiker AB, Parmar MKB, Spiegelhalter DJ, and the DAMOCLES study Group. Reported use of data monitoring committees in the main published reports of randomized controlled trials: a cross-sectional study. Clin Trials 2004; 1(1): 48-59.

22. Sacristán JA, Aguarón A, Avendaño-Solá C, et al. Patient involvement in clinical research: why, when, and how. Patient preference and adherence. 2016;10:631-640. doi:10.2147/PPA.S104259.

23. Taichman DB, Backus J, Baethge C, et al. Sharing clinical trial data: a proposal from the International Committee of Medical Journal Editors. The Lancet 2016.

24. Schulz, K.F., Altman, D.G., and Moher, D. CONSORT 2010 statement: updated guidelines for reporting parallel group randomised trials. BMJ 2010; 340: c332

25. ClinicalTrials.gov protocol data element definitions (draft): August 2011. ClinicalTrials.gov. http://prsinfo.clinicaltrials.gov/definitions.html. Accessed November 4, 2011

For peer review only

BMJ Open

A cross-sectional analysis of UK research studies in 2015: results from a scoping project with the UK Health Research Authority

Journal:	*BMJ Open*
Manuscript ID	bmjopen-2018-022340.R1
Article Type:	Research
Date Submitted by the Author:	22-May-2018

Complete List of Authors:	Clark, Tim; Ludwig-Maximilians University, Institut für Medizinische Informationsverarbeitung, Biometrie und Epidemiologie (IBE), Faculty of Medicine<br>Wicentowski, Richard; Swarthmore College, Computer Science Department<br>Sydes, Matthew; University College London, MRC Clinical Trials Unit, Institute of Clinical Trials and Methodology

**Primary Subject Heading**:	Research methods
Secondary Subject Heading:	Epidemiology
Keywords:	Clinical trials < THERAPEUTICS, STATISTICS & RESEARCH METHODS, Protocols & guidelines < HEALTH SERVICES ADMINISTRATION & MANAGEMENT

SCHOLARONE™ Manuscripts

Title: A cross-sectional analysis of UK research studies in 2015: results from a scoping project with the UK Health Research Authority

Running Head: Research in the UK

Version + Date: Version 3.00; (30-Aug-2018) -- post-acceptance

Tables/Figures: Tables=5

Figures=0

Name	Affiliation & IDs
Tim Clark	Institut für Medizinische Informationsverarbeitung, Biometrie und Epidemiologie (IBE), Faculty of Medicine, Ludwig-Maximilians University, Munich, Germany
Richard H Wicentowski	Computer Science Department, Swarthmore College, Swarthmore, PA, USA<br>ResearcherID: D-1051-2018<br>ORCID: 0000-0002-1187-9466
Matthew R Sydes	MRC Clinical Trials Unit at UCL, Institute of Clinical Trials and Methodology, UCL, London, UK<br>ResearcherID: C-3373-2008<br>ORCID: 0000-0002-9323-1371

Correspondence: Matthew Sydes m.sydes@ucl.ac.uk

Research support: M Sydes acknowledges support from the UK Medical Research Council: MC_UU_12023/24

For peer review only

For peer review only - http://bmjopen.bmj.com/site/about/guidelines.xhtml

Word Count:

Section	V2.00 (by section)	V1.00 (by section)	Change
Abstract	299	281	+16
Introduction	462	442	+20
Methods	741	713	+28
Results	822	823	-1
Discussion	1136	1139	-3
Total	3177	3117	

(Word counts include headings and reference numbers)

For peer review only

ABSTRACT

Objectives: To determine whether data on research studies held by the UK Health Research Authority (HRA) could be summarised automatically with minimal manual intervention. There are numerous initiatives to reduce research waste by improving the design, conduct, analysis and reporting of clinical studies. However, quantitative data on the characteristics of clinical studies and the impact of the various initiatives are limited.

Design: Feasibility study, using 1 year of data

Setting: We worked with the HRA on a pilot study using research applications submitted for UK-wide ethical review. We extracted into a single dataset, information held in anonymised XML files by the Integrated Research Application System (IRAS) and the HRA Assessment Review Portal (HARP). Research applications from 2014-2016 were provided. We used standard text extraction methods to assess information held in free-text fields. We use simple, descriptive methods to summarise the research activities that we extracted.

Participants: Not applicable -- records-based study

Interventions: Not applicable

Primary and secondary outcome measures: Feasibility of extraction and processing

Results: We successfully imported 1,775 non-duplicate research applications from the XML files into a single database. Of these, 963 were randomised controlled trials, 812 were other studies. Most studies received a favourable opinion. There was limited patient and public involvement (PPI) in the studies. Most, but not all, studies were planned for publication of results. Novel study designs (e.g. adaptive and Bayesian designs) were infrequently reported.

Conclusions: We have demonstrated that the data submitted from IRAS to the HRA and its HARP system are accessible and can be queried for information. We strongly encourage the development of fully-resourced collaborative projects to further this work. This would aid understanding of how study characteristics change over time and across

For peer review only

For peer review only - http://bmjopen.bmj.com/site/about/guidelines.xhtml

therapeutic areas, as well as the progress of initiatives to improve the quality and relevance of research studies.

For peer review only

For peer review only - http://bmjopen.bmj.com/site/about/guidelines.xhtml

ARTICLE SUMMARY

Strengths and limitations of this study

- First study to draw information from HRA's HARP system into one searchable dataset
- Anonymised data from the HRA's HARP system can be interrogated with minimal manual intervention
- Feasibility study, so limited to data from 2015 only and excluding Phase I healthy volunteer studies
- Aligning the questions from the XML document was not straightforward.
- The search terms chosen for the free-text fields were not exhaustive and because it was unfeasible in a pilot to review all applications, the sensitivity and specificity of the text-mining methodology could not be calculated

For peer review only

INTRODUCTION

The need to improve the quality of clinical research increasingly understood.[1] With a particular focus on clinical studies, the "gold standard" for evidence-based medicine, there has been a noticeable push towards: improving study protocols;[1] developing and implementing newer methodologies, such as adaptive designs;[2] involving patients in the design, conduct and management of studies;[3] and ensuring that study results are quickly and accurately reported.[4]

In order to properly evaluate the current state of clinical research and changes over time, it is clearly necessary to have unfettered access to the research protocols and study results.[5,6] There are a number of limitations in accessing such information. Published evaluations of the state of clinical research are mainly based on the clinical study publication due to the difficulty in obtaining access to unpublished research protocols.[7] Research registers are incomplete for many jurisdictions and/or are, like study publications, limited in detail compared to the research protocol. For example, clinicaltrials.gov[8] has good coverage of clinical studies in North America, where registration of clinical trials is mandatory. The WHO International Clinical Trials Registry Portal[9] provides access to 15 different regional or national registries, but both have markedly less detail than in each study's research protocol. Published evaluations of the literature are likely affected by publication bias and there are often discrepancies between research publications and their underpinning protocols.[10]

The ability to analyse a large database of application forms, each of which contain very detailed information taken directly from the research protocol, would enable researchers to perform a detailed examination of the characteristics of clinical studies, particularly those necessary for generating reliable evidence. This would aid understanding of how study characteristics change over time and across therapeutic areas, as well as the progress of initiatives to improve the quality and relevance of clinical studies.[11-15] Therefore, we approached the UK Health Research Authority (HRA), which oversees

For peer review only - http://bmjopen.bmj.com/site/about/guidelines.xhtml

national ethical review of health research conducted within the National Health Service (NHS). With the support of the HRA and having signed the appropriate confidentiality declarations, we developed a pilot project to determine the feasibility of interrogating with minimal manual intervention the data contained in HARP (HRA Assessment Review Portal); HARP is a web based management information system used by the HRA for all research ethics applications that require NHS REC (National Health Service Research Ethics Committee) review and/or HRA Approval. We negotiated access to data on clinical studies submitted in 2015. The extract had personal data and organisational identifiers removed by HRA and did not include phase I healthy volunteer studies.

Our pilot focused on automatically summarising the characteristics of clinical studies, including therapeutic area, blinding, randomization, use of Independent Data Monitoring Committee (IDMC), patient and public involvement (PPI) in the research, dissemination of the study results, and use of new methodologies such as adaptive designs.

For peer review only

METHODS

The UK Health Research Authority (HRA) collects applications for Research Ethics Committee "approval" (a favourable opinion) through the Integrated Research Application System (IRAS)[16] and stores them in HARP. IRAS is a web-based system used to capture the information required by review bodies in the UK, including the Medicines and Healthcare Products Regulatory Agency and HRA. When the forms are completed, IRAS saves the results as an XML document. XML is a commonly-used file format that stores structured data in a plain text format. The XML document can be used to generate PDF copies of the required forms and to repopulate the online web tool so users can later edit their applications.

Once the appropriate permissions were agreed, the HRA provided us with anonymised applications for clinical studies submitted for review from 2014-2016, excluding phase I healthy volunteer studies. For this pilot, we wished to focus on the extraction of clinical study characteristics of interest from the PDF versions of the HRA forms underlying the IRAS application system. We were provided with an incomplete mapping between the questions on PDF and the data stored in the XML document, such that many questions relevant to our study were not immediately labelled; nor was it apparent which questions were effectively switched on and off for applicants in completing the system's initial filter questions for their application. Therefore, we reverse-engineered the structure of the data in IRAS to determine which XML tags were responsible for creating the content we wanted to extract from the XML files to study.

The IRAS tool has been updated over time; not all of applications were created using the same version. Although the majority (91%) of the XML documents were created with one version (the latest version of the tool in the dataset), 8 different versions were present in the dataset we received. We were not provided with the documentation detailing the ways in which the versions differed; we had access only to the latest version. Our inspection of the data indicated that the differences relevant to this pilot

For peer review only - http://bmjopen.bmj.com/site/about/guidelines.xhtml

study were minor and we corrected for these when they were detected; however, there may be some undetected differences where the distinction between versions was important for our study.

For our pilot project, we chose to collect information on: the number of randomised controlled trials (RCT); the trial phase; the therapeutic area; the number of clinical trials of investigational medicinal products (CTIMPs – trials that require registration with regulatory body); the research method used (e.g. RCT, feasibility/pilot study, blinding); the use of blinding; the use of systematic or formal literature reviews in planning the study; the use of adaptive or Bayesian designs; the use of a data monitoring committee; the plans for dissemination of findings; and the role of patient and public involvement (PPI) in aspects of the research. Success would be measured in terms of our ability to access the information and generate descriptive summaries with minimal need for manual data reviews.

The data for our target areas were either held in multiple choice questions that we could identify and summarize or in free-text fields from which information needed to be determined and extracted. For those questions, we pre-processed the text using Spacy (https://spacy.io/), a natural language processing (NLP) package for Python. This involved identifying sentence boundaries and performing morphological analysis to convert words into their canonical dictionary form. We then used regular expressions to match phrases we were interested in, ignoring differences relating to spacing, hyphenation and capitalization; roman numerals were standardized to Latin numbers. For example, when looking for evidence that a systematic review was either performed or an existing review used during the study planning process, we searched for phrases such as “systematic review”, “reviewed systematically”, “literature review”, “evidence review” and “evidence-based review” in the answers provided to the relevant questions on the form, or instances where these words were used near each other, where “near” was defined as being within 3 words. For adaptive designs, we employed the US Food

For peer review only

For peer review only - http://bmjopen.bmj.com/site/about/guidelines.xhtml

and Drug Administration (FDA) definition, namely “a study that includes a prospectively planned opportunity for modification”.[17] We extracted the sentence containing the target phrase as well as one sentence before and after into a separate document. These textural extracts were then reviewed by the authors to identify true and false positives.

The data extracted from the separate XML files were collated into one dataset and descriptive analyses of this dataset were done with Stata (College Station, TX).

For peer review only

RESULTS

We received (30-Jun-2016) the XML files for 1,814 application records submitted for ethical review during the period specified, and extracted by HRA from the IRAS system. Of these, 1659 (92%) were from 2015, 154 (9%) from 2014, and 1 was from 2016. Three records were corrupted and could not be processed. In discussion with HRA, we discarded some records to remove duplicates. We kept one of 4 entries for the WHEAT trial which had been sent to multiple committees as part of research on consistency of Research Ethics Committee (REC) opinions.[18] We discarded one of two entries for a further study, which was initially given a favourable opinion and then submitted additionally to a specialist REC in Scotland (again, favourably). Thirty-two further records were discarded that initially had an unfavourable opinion before re-submission as a (near) identical, separate application for review. We discarded the first applications and kept the re-submissions, regardless of the subsequent review's outcome. Therefore, our dataset included 1,775 studies.

The filter questions switched off, for some applications, questions in which we were interested. For example, trial phase was only recorded for CTIMPs and the use of data monitoring committees was infrequently recorded. Furthermore, the use of blinding was not sufficiently well captured in the system to allow us to present reliable data.

A total of 963/1775 (54%) of the applications were stated as being randomised controlled trials (RCT). **Table 1** describes the disease setting, research method, CTIMP and REC opinion by whether they were an RCT. The most common research area, using the categorisation of the IRAS system, was cancer, followed by cardiovascular, musculoskeletal and respiratory diseases; around one third of records specified more than one area. This is broadly consistent with previous reviews.[19,20] Around one fifth of the RCTs was a pilot study; one third of records were employing more than one method. The REC gave a favourable opinion to the vast majority of applications (1740/1775, 98%), but required additional information for most of these 1323/1740 (75%).

For peer review only - http://bmjopen.bmj.com/site/about/guidelines.xhtml

Over half of the RCTs (515/963, 53%) were CTIMPs, of which 315/515 (61%) were CTIMPs of unlicensed products, 146/515 (28%) licensed CTIMPs in a new setting and 66/515 (13%) CTIMPs used according to their summary of product characteristics (SmPC); the categories were not mutually exclusive. Over a third (284/812, 35%) of the other studies (not RCTs) were also CTIMPs.

Table 2 describes the plans in design and dissemination. Most RCTs (895/963, 93%) were not recorded as being preceded by a formal systematic review of the literature. Only 15 (2%) RCTs were detectably designed under a Bayesian framework; 20 (2%) were detectable as having an adaptive design. There were 34 cluster randomised and 5 stepped wedge trials, overall. 614 (63%) RCTs were requesting new biological samples; 120 (12%) RCTs were seeking access to previous biological samples.

The large majority of research (1667/1775, 94%) was planned for dissemination in the peer-reviewed literature and most (1585/1775, 89%) were planned for conference presentations. A total of 475/963 (49%) RCTs and 352/812 (43%) other studies were planned for submission to regulatory authorities. A small number, 114/1775 (6%) were planning to offer raw data to external applicants as a key form of dissemination.

Table 3 notes the reported involvement of patients and the public (PPI) using the categories specified by IRAS. 726/1775 (41%) of studies claim PPI involvement in the design and 659/1775 (37%) planned PPI involvement in dissemination of the findings. PPI engagement in undertaking and management of the research was less common, and few studies (147/1775, 8%) planned PPI involvement in analysis. Around one third of studies involve PPI in two or more of these IRAS-defined areas.

Table 4 shows within CTIMPs the similarities and differences across the phase of research. Trial phase was asked as a series of separate yes/no questions for phase I, II,

For peer review only - http://bmjopen.bmj.com/site/about/guidelines.xhtml

III and IV. These were not mutually exclusive. For the purpose of summarising, we selected the highest level if more than one option was selected. Early phase trials were more likely to be designed within a Bayesian framework; phase III trials most often reported the use of a placebo and the use of an Independent Data Monitoring Committee; phase IV trials were least likely to be submitted to a regulatory authority or put on a website, but most often reported PPI involvement in all 5 broad areas.

Table 5 shows the outcome of the manual review of the extracted free-text fields. Accuracy, defined as number of true positives divided by overall total ranged from 43% for adaptive designs to 100% for stepped wedge. The false positives related to studies described as “phase 1/2" or “phase 2/3”, but with no evidence of an adaptive step, e.g. Phase 1/2 was often used to describe a classical pharmacokinetic study in patients rather than healthy volunteers; the database did not contain healthy volunteer studies. For “systematic review” and “meta-analysis” the search often picked up references to previous studies and not the planned study.

For peer review only

For peer review only - http://bmjopen.bmj.com/site/about/guidelines.xhtml

DISCUSSION

We achieved our primary feasibility aim of negotiating access to the centralised UK approvals system and devising a way of extracting information from a series of separate XML files. We anticipate that the programmes we developed could extract further pertinent points of information with minimal manual involvement. We were also able to achieve our primary descriptive aim of systematically reporting on the state of clinical research in the UK over around one year, focused on 2015.

We found that nearly one thousand RCTs were submitted for approval in the UK during that period, and more than 800 other studies. A key message is the volume of research activity in the UK, with the country demonstrably research active. The majority of applications received a favourable opinion but most required further information first, suggesting that applications could be better completed, saving time and effort for all parties.

It was notable that the reported use of adaptive designs was low and was not markedly different from earlier estimates.[19,20] This is disappointing, as many members of this design family have scope to reduce time to answer and/or reduce the average cost per answer, particularly by moving away early from treatment approaches that are not likely to improve outcomes sufficiently for patients.[21] However, Bayesian approaches have penetrated 10% of early phase CTIMPs. The reported use of Independent Data Monitoring Committees (IDMCs) for phase III CTIMPs was, perhaps, low at 58% but this is a substantial increase over the prevalence in trials results papers published in high impact-factor journals in 1990 and 2000.[22] Involvement of patients and the public in various aspects of research is increasingly seen as important, but reported rates of engagement were quite low: more than half of studies had no PPI involvement.[23] Cursory review of the free-text fields associated with these PPI categories suggests that some applicants may have been a little generous in choosing to select a particular category, suggesting that the numbers we report may be over-estimates. Other

For peer review only - http://bmjopen.bmj.com/site/about/guidelines.xhtml

comments suggest that PPI is not needed, perhaps reflecting that the value of PPI is yet to be recognised by some researchers.[3] Further research in this area is required.

Most but not all studies were planned for publication; there is still some way to go on this transparency aim. That raw data would be made available for some studies is encouraging and we hope that this will increase with time for appropriate projects from qualified researchers.[24]

Extracting the information from the HRA system was more effortful than expected. Moreover, important characteristics of clinical trials like blinding could not be analysed and certain search terms clearly had limitations. The issue of blinding is particularly important, as it is one of the desirable characteristics in generating reliable evidence from clinical trials.[11] However, now that we have completed this feasibility step, it is possible for future research projects to access an expanded dataset and work with the HRA to organise the database in a way that will facilitate its analysis. Access to several years of data would allow researchers, as well as the HRA itself, to examine the progress of initiatives to improve the quality of clinical research, such as those designed to encourage PPI and publication of clinical trial results.[13,15] We would also encourage the HRA to adopt standard definitions to describe the characteristics of clinical trials, as done in the USA.[25] Confusion over the definition of terms greatly complicates efforts to automatically analyse clinical research.

Our pilot work has some limitations. We had no straightforward way of aligning the questions in the PDF document with the information stored in the XML document. Perhaps if we had negotiated access to the backend software used by IRAS to generate these PDF forms, we may have been able to do this automatically; instead, we needed to reverse-engineer the IRAS system in order to figure out which XML tags were responsible for creating the content we wanted to study. Not all of the applications had been created using the same version of the IRAS tool.[16] Although a large majority of the

For peer review only

For peer review only - http://bmjopen.bmj.com/site/about/guidelines.xhtml

documents were created using the latest version of the tool, 8 different versions were present in the dataset we received. We did not receive any guidance on the ways in which one version of the tool differed from another version, and we only had access to the latest version. We made efforts to determine the specific situations in which questions were not asked rather than asked but not answered, in order to make this distinction ourselves, though ideally this could be indicated directly in the XML dataset. The labelling of missing answers and inactivated questions was not consistent. There is scope for applicants to misunderstand the questions and misrepresent the data. For example, 3 trials were noted as using RCT methodology but answered "no" to the question: "Will participants be allocated to groups at random?" We checked the data and found these were not actually RCTs; we included them with the "other studies". Likely this error by the applicants activated or de-activated some questions inappropriately. In this example, the RCT method question actually asked about randomisation of individuals and these three applicants who submitted cluster randomised trials had answered this question inconsistently. Finally, the search terms chosen for the free-text fields were not exhaustive and because it was unfeasible in a pilot to review all applications, the sensitivity and specificity of the NLP package could not be calculated.

Some interesting information was held only in the free-text fields. We used standard methods to extract this information using automation supplemented by manual checking. There is a wealth of free-text information, which through the application of text-mining techniques, could provide incredibly valuable insights into the characteristics of health research. For this pilot study, we did not have the resources to review all of the questions and we particularly could not spend time reading free-text fields. Therefore, we felt obliged not to correct categorical questions if we noticed they were contrasted by free-text; however, a fully-resourced study could do this. For example, we noticed at least one entry, which ticked boxes for PPI engagement and expanded on this by claiming that, as doctors, they knew what patients wanted and did not need to trouble patients for their time on these aspects.

For peer review only - http://bmjopen.bmj.com/site/about/guidelines.xhtml

In conclusion, we have demonstrated that anonymised data from the HRA's research system is accessible and can be queried for information. We strongly encourage the development of fully-resourced collaborative projects to delve more deeply into this data. We believe that it is imperative that the characteristics of clinical research in the UK are understood, as these underpin clinical guidance. Finally, there are many ongoing initiatives to improve the quality of clinical research, but only by fully understanding the state of, and changes to, the research profile of the UK, can we appreciate their impact.

For peer review only

AUTHOR CONTRIBUTIONS

Grouped by role

Conceptualised the study – TPC, MRS; Designed the study – TPC, RHW, MRS; Collected the data – TPC, RHW, MRS; Analysed and interpreted the data – TPC, RHW, MRS; Wrote critical sections of manuscript – TPC, RHW, MRS; Reviewed and approved final manuscript – TPC, RHW, MRS

Grouped by author

TPC conceptualised the study, designed the study, collected the data, analysed and interpreted the data, wrote critical sections of manuscript, and reviewed and approved final manuscript. RHW designed the study, collected the data, analysed and interpreted the data, wrote critical sections of manuscript, and reviewed and approved final manuscript. MRS conceptualised the study, designed the study, collected the data, analysed and interpreted the data, wrote critical sections of manuscript, and reviewed and approved final manuscript.

For peer review only

PATIENT AND PUBLIC INVOLVEMENT

There was no patient or public involvement in this records-based pilot study. There should be PPI engagement in any subsequent study.

For peer review only

DATA SHARING

The data used in this analysis were obtained from the Health Research Authority under a confidentiality agreement and is not under the researcher's gift to share. Applications for this data or expanded data must involve the HRA. Access to the processing code is available through the MRC Clinical Trials Unit at UCL's usual Data Release Request approach.

For peer review only

For peer review only - http://bmjopen.bmj.com/site/about/guidelines.xhtml

COMPETING INTERESTS

MRS = None

RHW = None

TPC = Worked as a consultant for the clinical research organisation ICON in the previous three years

For peer review only

ACKNOWLEDGEMENTS

Hazel Gage, Amanda Hunn, Janet Messer - UK Health Research Authority; Prof. Dr. Ulrich Mansmann, IBE, Munich, Germany.

For peer review only

TABLES AND FIGURES

Table 1: Overview of research activities submitted for REC approval

Characteristic	RCT N	RCT %	Other N	Other %	Total N	Total %
N	963		812		1775	
**Therapeutic area[1]**						
Cancer	168	17%	192	24%	360	20%
Cardiovascular	94	10%	94	12%	188	11%
Musculoskeletal	97	10%	30	4%	162	9%
Respiratory	97	10%	30	4%	159	9%
Paediatrics	56	6%	30	4%	148	8%
Neurological	73	8%	30	4%	145	8%
Mental Health	66	7%	30	4%	117	7%
Inflammatory & Immune System	72	7%	30	4%	113	6%
Oral and Gastrointestinal	64	7%	30	4%	113	6%
Blood	44	5%	67	8%	111	6%
Diabetes	58	6%	38	5%	96	5%
Infection	55	6%	30	4%	92	5%
Renal and Urogenital	39	4%	30	4%	82	5%
Generic Health Relevance	46	5%	30	4%	76	4%
Metabolic and Endocrine	32	3%	30	4%	70	4%
Dementias & Neurodegenerative	27	3%	36	4%	63	4%
Skin	36	4%	30	4%	57	3%
Reproductive Health & Childbirth	30	3%	30	4%	56	3%
Stroke	27	3%	30	4%	53	3%
Eye	24	2%	28	3%	52	3%
Injuries and Accidents	22	2%	30	4%	36	2%
Congenital Disorders	10	1%	20	2%	30	2%
Ear	6	1%	7	1%	13	1%
**Count of therapeutic areas claimed**						
None	52	5%	49	6%	101	6%
1	660	69%	495	61%	1155	65%
2	201	21%	200	25%	401	23%
3	29	3%	49	6%	78	4%
4 or more	21	2%	19	2%	40	2%
**Research methods used[1]**						
Randomised controlled trial	963	100%	2	0%	965	54%
Feasibility/ pilot study	177	18%	300	37%	477	27%
Questionnaire	98	10%	139	17%	237	13%
Cohort observation	23	2%	161	20%	184	10%
Qualitative research	90	9%	76	9%	166	9%
Controlled trial, no randomisation	3	0%	157	19%	160	9%
Laboratory study	30	3%	47	6%	77	4%
Case series/ case note review	7	1%	45	6%	52	3%
Cross-sectional study	4	0%	31	4%	35	2%
Case control	5	1%	25	3%	30	2%
Database analysis	8	1%	17	2%	25	1%
Epidemiology	7	1%	5	1%	12	1%
Meta-analysis	0	0%	0	0%	0	0%

For peer review only

For peer review only - http://bmjopen.bmj.com/site/about/guidelines.xhtml

Characteristic	RCT N	RCT %	Other N	Other %	Total N	Total %
Other	68	7%	269	33%	337	19%
**Count of research methods used**						
1	641	67%	522	64%	1163	66%
2	192	20%	160	20%	352	20%
3	73	8%	97	12%	170	10%
4	48	5%	25	3%	73	4%
5	7	1%	7	1%	14	1%
6	2	0%	1	0%	3	0%
**CTIMP**						
Unlicensed	315	33%	189	23%	504	28%
New use	146	15%	70	9%	216	12%
Within SmPC	66	7%	26	3%	92	5%
Other	10	1%	5	1%	15	1%
Any of these	*515*	*53%*	*284*	*35%*		
**Involves ionising radiation**						
Yes	666	69%	582	72%	1248	71%
No	297	31%	222	28%	519	29%
Missing	0	n/a	8	n/a	8	n/a
**REC opinion**						
Favourable	942	98%	798	98%	1740	98%
Unfavourable	21	2%	14	2%	35	2%
Favourable	200	21%	217	27%	417	23%
Favourable (extra info)	742	77%	581	72%	1323	75%
Unfavourable	21	2%	14	2%	35	2%

[1] Not mutually exclusive

For peer review only

Table 2: Issues in design and dissemination in all entries

Characteristic	RCT N	RCT %	Other N	Other %	Total N	Total %
N	963		812		1775	
**Review of data as part of development**						
Neither	895	93%	759	93%	1654	93%
Systematic review only	38	4%	32	4%	70	4%
Meta-analysis only	18	2%	13	2%	31	2%
Both	12	1%	8	1%	20	1%
**Design characteristics**						
Neither	926	96%	778	96%	1704	1%
Adaptive design	20	2%	9	1%	29	2%
Bayes design	15	2%	24	3%	39	2%
Both	2	0%	1	0%	3	0%
Cluster randomised	26	3%	8	1%	34	2%
Stepped wedge	3	0%	2	0%	5	0%
**Sample collection**						
Taking new samples	614	64%	422	52%	1036	58%
Accessing stored samples	120	12%	110	14%	230	13%
**Plans for dissemination[2]**						
Peer reviewed scientific journal	912	95%	755	93%	1667	94%
Conference presentation	869	90%	716	88%	1585	89%
Internal report	641	67%	526	65%	1167	66%
Publication on website	536	56%	367	45%	903	51%
Submission to regulatory authority	475	49%	352	43%	827	47%
Other publication	239	25%	178	22%	417	23%
Access to raw data	191	20%	133	16%	114	6%
Other	0	0%	3	0%	324	18%
No plans to report or disseminate					3	0%

[1] Not asked for non-regulatory RCTs

[2] Not mutually exclusive

For peer review only

380 **Table 3: Reported patient and public involvement**

Characteristic	RCT N	RCT %	Other N	Other %	Total N	Total %
N	963		812		1775	
**Areas of PPI activity[1]**						
Design of the research	403	42%	323	40%	726	41%
Dissemination of findings	375	39%	284	35%	659	37%
Undertaking the research	242	25%	219	27%	461	26%
Management of the research	225	23%	129	16%	354	20%
Analysis of results	72	7%	75	9%	147	8%
**Number of areas of PPI activity**						
None	389	40%	302	37%	691	39%
1	205	21%	209	26%	414	23%
2	139	14%	117	14%	256	14%
3	125	13%	93	11%	218	12%
4	66	7%	37	5%	103	6%
All	39	4%	32	4%	71	4%

381 [1] Not mutually exclusive

For peer review only

Table 4: Characteristics of the CTIMPs by trial phase

Trial phase[1] / Characteristic	Ph I N	%	Ph II N	%	Ph III N	%	Ph IV N	%	Total N	%
N – all entries	99		287		324		98		808	
**Design characteristics**										
Neither	86	89%	261	93%	303	94%	90	94%	740	93%
Adaptive	1	1%	7	2%	13	4%	3	3%	24	3%
Bayes	10	10%	12	4%	5	2%	2	2%	29	4%
Both	0	0%	2	1%	0	0%	1	1%	3	0%
Cluster randomised	0	0%	0	0%	0	0%	0	0%	0	0%
Stepped wedge	0	0%	0	0%	0	0%	0	0%	0	0%
Involves placebo	21	22%	123	44%	163	51%	25	26%	332	42%
**Independent Data Monitoring Committee**										
No	40	82%	84	59%	61	42%	46	78%	231	58%
Yes	9	18%	59	41%	84	58%	13	22%	165	42%
Missing	*8*		*139*		*176*		*37*		*400*	
**Methods of dissemination**										
Peer reviewed scientific journal	86	89%	265	94%	290	90%	93	97%	86	89%
Submission to regulatory authority	85	88%	215	76%	272	85%	48	50%	85	88%
Conference presentation	82	85%	253	90%	271	84%	89	93%	82	85%
Internal report	71	73%	212	75%	259	81%	60	63%	71	73%
Publication on website	56	58%	161	57%	203	63%	45	47%	56	58%
Other publication	26	27%	76	27%	92	29%	17	18	26	27%
Access to raw data	8	8%	12	4%	25	8%	8	8%	8	8%
Other	12	12%	47	17%	48	15%	18	19%	12	12%
No plans to report or dissemination	0	0%	0	0%	2	1%	0	0%	0	0%

For peer review only

Trial phase[1] / Characteristic	Ph I N	Ph I %	Ph II N	Ph II %	Ph III N	Ph III %	Ph IV N	Ph IV %	Total N	Total %
**Areas of PPI activity[2]**										
Design of the research	26	27%	82	29%	39	12%	50	52%	26	27%
Management of the research	10	10%	34	12%	28	9%	31	32%	10	10%
Undertaking the research	14	14%	47	17%	36	11%	29	30%	14	14%
Analysis of results	1	1%	15	5%	7	2%	12	13%	1	1%
Dissemination of findings	20	21%	74	26%	64	20%	44	46%	20	21%
None of the above	53	55%	149	53%	223	69%	35	36%	460	58%
**REC opinion**										
Favourable	97	98%	277	97%	318	98%	93	95%	785	97%
Unfavourable	0	0%	5	2%	3	1%	3	3%	11	1%
Favourable	14	14%	51	18%	40	12%	15	15%	120	15%
Favourable (after extra info)	83	84%	226	79%	278	86%	78	80%	665	82%
Unfavourable	0	0%	5	2%	3	1%	3	3%	11	1%

[1] Trial phase calculated

[2] Not mutually exclusive

For peer review only - http://bmjopen.bmj.com/site/about/guidelines.xhtml

Table 5: Review of possible search terms

Design element / Search term	Performance of search term[1] – At sentence level: True Positive	False Positive	Accuracy	At study level: True Positive	False Positive	Accuracy
**Systematic review**						
Review [near] systematically	2	0	100%	2	0	100%
Evidence [near] review	4	1	80%	4	1	80%
Systematic [near] review	98	39	72%	58	23	72%
Literature [near] review	29	13	69%	26	12	68%
Evidence-based review	*0*	*0*	*n/a*	*0*	*0*	*n/a*
**Summary[1,2]**	**133**	**53**	**72%**	**90**	**36**	**71%**
**Meta-analysis**						
Pooled analysis	2	0	100%	2	0	100%
Meta-analysis	77	31	71%	49	19	72%
Integrated analysis	*0*	*0*	*n/a*	*0*	*0*	*n/a*
**Summary[1,2]**	**79**	**31**	**72%**	**51**	**19**	**73%**
**Adaptive design**						
Adaptive design	19	0	100%	13	0	100%
Adaptive randomisation	8	0	100%	5	0	100%
Continual reassessment	2	0	100%	1	0	100%
Sample size re-estimation	15	0	100%	9	0	100%
Seamless design	1	0	100%	1	0	100%
Phase 1/2	4	43	9%	3	37	8%
Phase 2/3	2	25	7%	2	23	8%
CRM design	*0*	*0*	*n/a*	*0*	*0*	*n/a*
Drop-the-loser	*0*	*0*	*n/a*	*0*	*0*	*n/a*
MAMS design	*0*	*0*	*n/a*	*0*	*0*	*n/a*
Multi-arm multi-stage	*0*	*0*	*n/a*	*0*	*0*	*n/a*
Pick-the-winner	*0*	*0*	*n/a*	*0*	*0*	*n/a*
**Summary[1,2]**	**51**	**68**	**43%**	**31**	**60**	**34%**
**Bayes**						
Bayes	83	2	98%	42	1	98%
**Summary[1,2]**	**83**	**2**	**98%**	**42**	**1**	**98%**
**Cluster RCT**						
Cluster RCT	26	0	100%	11	0	100%
Control cluster	6	0	100%	4	0	100%
Randomised cluster	2	0	100%	1	0	100%
Cluster random	46	1	98%	30	1	97%
**Summary[1,2]**	**80**	**1**	**99%**	**34**	**1**	**97%**
**Stepped wedge**						
Step [near] wedge	1	0	100%	1	0	100%
Stepped [near] wedge	16	0	100%	5	0	100%
**Summary[1,2]**	**17**	**0**	**100%**	**5**	**0**	**100%**

NB The table reports on whether each search term accurately identified a specific design element of the study in selected free-text fields. All variations of the spelling, capitalisation, hyphenation etc., were covered in the search terms used.

[1] The left side of the table separately considers each sentence which matched a search term accurately identified the design property. There were 613 such sentences. The Summary row in each subsection is the sum of the constituent search terms.

[2] The right side of the table separately considers whether any sentence in a study matching a search term accurately identified the design property. Some studies matched a search term repeatedly, therefore the numbers are

For peer review only

smaller. The Summary row in each subsection reflects a search across **all** of its constituent search terms. Note that some studies matched more than one search term in a subsection, so the Summary row may not be the sum of the rows above e.g. a study that uses both "cluster random" and "control cluster" would appear only once in the Summary row, and would appear separately in the "cluster random" and "control cluster" rows.

For peer review only - http://bmjopen.bmj.com/site/about/guidelines.xhtml

REFERENCES

1. Ioannidis JP. Why Most Clinical Research Is Not Useful. *PLoS Med* 2016; **13**(6): e1002049.
2. Hatfield I, Allison A, Flight L, Julious SA, Dimairo M. Adaptive designs undertaken in clinical research: a review of registered clinical trials. *Trials* 2016; **17**(1): 150.
3. Elliott J. HRA Strategy for Public Involvement (v1.1). London, UK: Health Research Authority, 2013.
4. alltrials.net. alltrials.net (accessed 2013-05-24 2013).
5. English R, Lebovitz Y, Griffin R, Forum on Drug Discovery Development and Translation, Institute of Medicine. Transforming Clinical Research in the United States: Challenges and Opportunities: Workshop Summary. Institute of Medicine of the National Acadamies, Naitonal Acadamies Press; 2010. p. 151.
6. Chalmers I, Glasziou P, Godlee F. All trials must be registered and the results published. *BMJ* 2013; **346**: f105.
7. Chan AW, Upshur R, Singh JA, Ghersi D, Chapuis F, Altman DG. Research protocols: waiving confidentiality for the greater good. *BMJ* 2006; **332**(7549): 1086-9.
8. ClinicalTrials.gov. www.clinicatrials.gov. (accessed 2018-05-22.
9. Organisation WH. WHO Clinical Trials Registration Portal. http://www.who.int/ictrp/en/ (accessed 2018-05-22.
10. Chan AW, Hrobjartsson A, Jorgensen KJ, Gotzsche PC, Altman DG. Discrepancies in sample size calculations and data analyses reported in randomised trials: comparison of publications with protocols. *BMJ* 2008; **337**: a2299.
11. Califf RM, Zarin DA, Kramer JM, Sherman RE, Aberle LH, Tasneem A. Characteristics of clinical trials registered in ClinicalTrials.gov, 2007-2010. *JAMA : the journal of the American Medical Association* 2012; **307**(17): 1838-47.
12. Grignolo A. The Clinical Trials Transformation Initiative (CTTI). *Ann Ist Super Sanita* 2011; **47**(1): 14-8.
13. INVOLVE. Public involvement in research: values and principles framework. Eastleight, 2015.
14. Chan AW, Tetzlaff JM, Altman DG, Dickersin K, Moher D. SPIRIT 2013: new guidance for content of clinical trial protocols. *Lancet* 2013.
15. Moorthy VS, Karam G, Vannice KS, Kieny MP. Rationale for WHO's new position calling for prompt reporting and public disclosure of interventional clinical trial results. *PLoS Med* 2015; **12**(4): e1001819.
16. Health Research Authority. IRAS -- Integrated Research Application System. https://www.myresearchproject.org.uk/ (accessed 2018-05-22.
17. FDA. Draft Guidance for Industry Adaptive Design Clinical Trials for Drugs and Biologics 2010.
18. Gale C, Hyde MJ, Modi N, group Wtd. Research ethics committee decision-making in relation to an efficient neonatal trial. *Arch Dis Child Fetal Neonatal Ed* 2017; **102**(4): F291-F8.
19. Clark T. Sample size calculations in clinical trials of investigational medicinal products: an analysis of ongoing practice. *Der Andere Verlag* 2013.

For peer review only

For peer review only - http://bmjopen.bmj.com/site/about/guidelines.xhtml

20. Clark T, Berger U, Mansmann U. Sample size determinations in original research protocols for randomised clinical trials submitted to UK research ethics committees: review. *BMJ* 2013; **346**(mar21 1): f1135-f.

21. Parmar MK, Barthel FM, Sydes M, et al. Speeding up the evaluation of new agents in cancer. *J Natl Cancer Inst* 2008; **100**(17): 1204-14.

22. Sydes MR, Altman DG, Babiker AB, Parmar MKB, Spiegelhalter DJ, and the DAMOCLES study Group. Reported use of data monitoring committees in the main published reports of randomized controlled trials: a cross-sectional study. *Clin Trials* 2004; **1**(1): 48-59.

23. Sacristan JA, Aguaron A, Avendano-Sola C, et al. Patient involvement in clinical research: why, when, and how. *Patient Prefer Adherence* 2016; **10**: 631-40.

24. Taichman DB, Backus J, Baethge C, et al. Sharing clinical trial data: a proposal from the International Committee of Medical Journal Editors. *The Lancet* 2016.

25. ClinicalTrials.gov. ClinicalTrials.gov Protocol Registration Data Element Definitions for Interventional and Observational Studies. 2017. https://prsinfo.clinicaltrials.gov/definitions.html (accessed 2018-05-22.

For peer review only

For peer review only - http://bmjopen.bmj.com/site/about/guidelines.xhtml
